# A History of Endometriosis Is Associated With Decreased Peripheral NK Cytotoxicity and Increased Infiltration of Uterine CD68^+^ Macrophages

**DOI:** 10.3389/fimmu.2021.711231

**Published:** 2021-08-31

**Authors:** Linlin Wang, Longfei Li, Yuye Li, Chunyu Huang, Ruochun Lian, Tonghua Wu, Jingwen Ma, Yan Zhang, Yanxiang Cheng, Lianghui Diao, Yong Zeng

**Affiliations:** ^1^ Shenzhen Key Laboratory of Reproductive Immunology for Peri-implantation, Shenzhen Zhongshan Institute for Reproduction and Genetics, Shenzhen Zhongshan Urology Hospital, Shenzhen, China; ^2^ Department of Obstetrics and Gynecology, Renmin Hospital of Wuhan University, Wuhan, China; ^3^ Laboratory of Molecular Pharmacology, Department of Pharmacology, School of Pharmacy, Southwest Medical University, Luzhou, China; ^4^ Department of Paediatrics & Adolescent Medicine, Li Ka Shing Faculty of Medicine, University of Hong Kong, Hong Kong, Hong Kong, SAR China

**Keywords:** endometriosis, estradiol, peripheral natural killer cytotoxicity, uterine macrophages, reproductive failure

## Abstract

Women with endometriosis may have a defective immune system. However, evidence of the immune responses of endometriosis patients with a history of endometriosis surgery is lacking, and the association between the location of endometriosis lesions and immune responses is unclear. This retrospective study included 117 females with reproductive failure and a history of endometriosis and 200 females with reproductive failure but without endometriosis to analyze their endometrial and peripheral immune responses. The results show that endometriosis was associated with decreased peripheral natural killer (NK) cytotoxicity and increased uterine macrophages. Peripheral NK cytotoxicity at effector-to-target ratios of 25:1 and 50:1 was significantly reduced in women with a history of endometriosis from that of the control group (26.6% *versus* 33.3% and 36.1% *versus* 43.3%, respectively, both *P* < 0.001). Furthermore, after further division of patients into three subgroups according to the location of endometriosis lesions, we observed that NK cytotoxicity in the endometriosis subgroups, especially the mixed endometriosis group, was strongly decreased from that of the controls (*P* = 0.001). The endometrial CD68^+^ macrophage proportion in the mixed endometriosis subgroup was higher than that in the control group (2.8% *versus* 2.1%, *P* = 0.043). In addition, the baseline estradiol (E2) level was weakly correlated with the percentage of endometrial macrophages (r = 0.251, *P* = 0.009), indicating a potential association among the endocrine system, endometrial immune environment, and endometriosis. This study indicated that peripheral NK cytotoxicity and endometrial immune cell profiles could be useful for diagnosing and treating endometriosis and endometriosis-related reproductive diseases.

## Introduction

Endometriosis, defined as the presence of endometrial-like tissue outside the uterus, is a common estrogen-dependent gynecological disease with an overall prevalence of 10% in women of reproductive age (1). Endometriosis is a risk factor for many obstetrical and gynecological disorders, and leads to infertility in approximately 30% of affected women (2). Moreover, endometriosis has a high recurrence rate (3). However, the pathogenesis of endometriosis is still unclear, which poses considerable obstacles to its diagnosis and treatment.

According to the location of endometriotic lesions, endometriosis is divided into deep infiltrating endometriosis, ovarian endometriosis (chocolate cysts), and peritoneal endometriosis (pelvic endometriosis) (4). To date, the molecular mechanisms governing the initiation and development of ectopic endometriotic lesions remain controversial. Along with retrograde menstruation, endometrial stem cell implantation, Müllerian remnant abnormalities, and coelomic metaplasia, the pathogenesis of endometriosis may also involve the dysregulation of estrogen and progesterone, angiogenesis or vasculogenesis, immune system, cytokines, and chemokines (5). Most importantly, systemic and local immune responses may play a primary role in the pathophysiology and symptomatology of endometriosis (6, 7). It is speculated that women with endometriosis may have a defective immune system, with endometrial fragments themselves acquiring the ability to evade immune surveillance and clearance (6).

Previous studies have reported changes in the composition or functions of immune cells, including macrophages (8), natural killer (NK) cells (9), dendritic cells (DC) (10–12), and T (13–15) and B cells (15, 16) in both the eutopic or ectopic endometrium or the peripheral blood of women with endometriosis. However, the composition or functions of those immune cells remain controversial. Additionally, evidence regarding the immune responses of endometriosis patients with a history of surgery for endometriosis is lacking, and the association between the location of endometriosis lesions and patient immune responses is unclear.

Therefore, in order to verify the endometrial and peripheral immune responses in patients with a history of endometriosis, this study retrospectively analyzes various immune parameters in 117 females with reproductive failure and a history of endometriosis and 200 females with reproductive failure but without endometriosis. The former group are further divided into three subgroups, including ovarian, pelvic, and mixed endometriosis, to determine the effect of the specific location of endometriosis on immune responses. Evaluating the immune response is important for revealing the pathogenesis of endometriosis and endometriosis-related reproductive failure. It can also facilitate the development of innovative diagnostics and therapeutics for associated diseases.

## Materials and Methods

### Study Population

This retrospective study was evaluated and approved by the ethics committee of Shenzhen Zhongshan Urology Hospital (Approval number: SZZSECHU-F-2019040). Initially, this study included 682 females who visited the Reproductive Immunology Department of Shenzhen Zhongshan Urology Hospital from January 2014 to October 2019, who had a history of repeated implantation failure (RIF), recurrent miscarriage (RM), or other cases of reproductive failure. RIF is defined as women who experienced two or more retrieval cycles and transferred more than 10 high-quality embryos (17) without pregnancy (18). RM is defined as patients who experienced two or more miscarriages before week 20 of gestation. Other cases of reproductive failure refer to patients who failed to achieve a pregnancy or experienced miscarriage but did not meet the standard of RIF or RM. Patients with hormonal or metabolic disorders, known clinical autoimmune disease, chronic endometritis, uterine, tubal or ovary abnormality, adenomyosis (n = 265), or lacking baseline data (n = 100) were excluded from this study. Finally, the analysis included 317 patients, including 117 women with a history of endometriosis and 200 women without endometriosis as the control group. Patients in the endometriosis group were diagnosed with endometriosis through laparoscopy, B-scan ultrasonography, and observations of the symptoms of endometriosis such as pelvic pain, infertility, pain after sex, and heavy menstrual periods. All patients in the endometriosis group were without endometriosis on the date of sampling according to an absence of the symptoms of endometriosis and B-scan ultrasonography analysis, with or without laparoscopy. On the other hand, patients in the control group were defined by their absence of endometriosis symptoms and B-scan ultrasonography, with or without laparoscopy. According to lesion locations, the endometriosis group was further divided into three subgroups, including ovarian (n = 44), pelvic (n = 57), and mixed endometriosis (n = 16, patients diagnosed with both ovarian and pelvic endometriosis).

### Endometrial Preparation Program

Hormone replacement therapy cycles or natural cycles were used to collect the endometrium from the patients. During natural cycles, progesterone (Dydrogesterone Tablet, Solvay Pharmaceuticals, Netherlands) was administered at a dose of 20 mg on day 18 of the cycle, then continued until the mid-luteal phase of the menstrual cycle. During hormone replacement therapy cycles, estrogen and progesterone were used consecutively to mimic the endocrine conditions of the endometrium during the natural cycle. Estradiol valerate (Delpharm Lille SAS, Bayer AG, Berlin, Germany) was administered during the cycle as follows: 4 mg on day 3–7, 6 mg on day 8–12, 8 mg on day 13 to the day of endometrial sampling. Progesterone (Progesterone Injection, Zhejiang Xianju Pharmaceutical Co. Ltd. Zhejiang, China) was administered at a dose of 60 mg on day 18 of the cycle, then continued until the mid-luteal phase of the menstrual cycle.

### Measurement of Serum Hormone Levels

Serum hormones were all collected on day three of the menstrual cycle. The concentrations of estradiol (E2), progesterone (P), follicle-stimulating hormone (FSH), luteinizing hormone (LH), prolactin (PRL), and testosterone (T) in the sera of the patients were measured with Elecsys Estradiol III, progesterone II, FSH, LH, Prolactin II, and Testosterone II kits, respectively (Elecsys, Olathe, USA), and analyzed in an immunology analyzer (Roche Diagnostics, Basel, Switzerland).

### Endometrial Immunological Parameters

Endometrial tissue samples were collected during the mid-luteal phase (LH day 7–9) of the menstrual cycle. Samples were fixed and embedded in paraffin wax for immunohistochemical staining. Samples were stained in an auto-immunostainer (Leica Bond III system, Germany) using a Bond polymer refine detection kit (Leica Microsystems, Germany). All staining batches included appropriate controls and employed commercially available monoclonal antibodies for CD56, CD68, CD1a, CD83, CD57, CD8, and FoxP3, and the immunohistochemical protocols outlined in **Supplementary Table S1**.

The samples were first scanned at low magnification. Then, images of 30 random fields (10.68 mm^2^) per section were captured in high-power fields (HPF, magnification 200×). All immune cell populations from each sample were characterized and quantified using the cell segmentation and phenotype cell tool of InForm^®^ Cell Analysis™ software (Perkin Elmer, Waltham, MA, USA) under the supervision of two pathologists. The ratio of each immune cell population was assessed as a percentage on all endometrial cells in 30 randomly selected high-power fields (HPF, magnification 200×).

### Peripheral Immunological Parameters

Peripheral blood samples were collected during the mid-luteal phase (LH day 7–9) of the menstrual cycle for analyses of the peripheral immune responses, including lymphocytes subsets, NK cytotoxicity, NK cell surface receptors and intracellular mediators, and intracellular cytokines of peripheral CD4^+^ T cells.

A Multitest 6-color TBNK Reagent Kit (BD Biosciences, San Jose, CA, USA) was used to quantify the percentages of T, B, and NK lymphocytes in 100-μl erythrocyte-lysed whole blood. Anti-CD45-PerCP-Cy5.5, anti-CD3-FITC, anti-CD4-PE-Cy7, anti-CD8-APC-Cy7, anti-CD19-APC, and anti-CD16/CD56-PE, (all from BD Biosciences, San Jose, CA, USA) were used. The staining cells were then analyzed on a BD FACSCanto II flow cytometer (BD Biosciences, San Jose, CA, USA).

NK cell cytotoxicity was evaluated by K562 target-killing assay with peripheral blood mononuclear cells (PBMC) isolated from the patients. K562 cells purchased from the China Center for Type Culture Collection were washed in PBS and dyed with 3,3-dioctadecyloxacarbocyx acarbocyanine perchlorate-FITC (Sigma-Aldrich, St. Louis, MO, USA). PBMCs were isolated by density gradient centrifugation using Ficoll-Paque (Amersham Bioscience, Shanghai, China). Subsequently, PBMCs and K562 cells were cocultured at effector-to-target ratios of 25:1 (ET25) and 50:1 (ET50) at 37°C in a 5% CO_2_ humidified incubator. After 4 h incubation, propidium iodide-PE (Sigma-Aldrich, St. Louis, MO, USA) was added to determine the proportion of dead K562 cells by flow cytometry.

To evaluate the NK cell surface receptors and intracellular mediators, isolated PBMCs were stained with CD3‐PerCP and CD56‐PE‐Cy7 to characterize the NK cells. NK cell surface receptors were stained with NKG2D‐FITC, NKp30‐PE, NKp46‐APC, CD158a‐FITC, and CD158b‐PE for 30 min at room temperature in the dark, then detected by flow cytometry. Intracellular proteins were stained with granzyme B‐PE, perforin‐Alexa Fluor 647, and granulysin‐Alexa Fluor 647 for 30 min at room temperature in the dark, then detected by flow cytometry.

For the intracellular cytokine staining of peripheral CD4^+^ T cells, fresh blood (500 μL) was stimulated with 10 ng/mL phorbol myristate acetate (PMA; Sigma‐Aldrich, USA) and 1.25 μg/mL ion‐omycin (Sigma) in the presence of 10 μg/mL brefeldin A (BFA) for 4 h at 37°C in 5% CO_2_. One hundred microliters of stained blood was aliquoted into a new flow tube. After removing red blood cells, samples were permeabilized with FACS Permeabilizing Solution (BD Bioscience), then stained for CD3‐PerCP, CD8‐APC‐Cy7, TNF‐α‐APC, IFN‐γ‐FITC, or matched isotype controls (BD Bioscience) in the dark for 30 min.

### mRNA Expression of Estrogen Receptors and CD68 *via* EndometDB

EndometDB is an interactive web-based user interface for browsing the gene expression database of collected samples without the need for computational skills. EndometDB incorporates the expression data from 115 endometriosis patients and 53 controls, with over 24,000 genes and clinical features. The mRNA expression of estrogen receptor 1 (ESR1), estrogen receptor 2 (ESR2), and CD68 in the endometrium and peritoneum of the control group, and in the eutopic and ectopic endometria and peritoneum of patients with endometriosis were analyzed in EndometDB (https://endometdb.utu.fi/gene_analysis/) (19). Specifically, all clinical and sample data in the database were selected, and the patients were separated by age. Box plots (parameter settings: combine lesions, use log2-scale, show legend, and display sample counts) were used to show the data. Pearson correlation was used to show the correlation matrix between ESR1, ESR2, and CD68 *via* EndometDB.

### Statistical Analysis

Statistical analyses were performed using SPSS for Windows (version 25.0, SPSS Inc., Chicago, IL). The distribution of continuous data was checked for normality using the Kolmogorov-Smirnov test. Continuous variables with a normal distribution were analyzed by the student’s t-test or one-way ANOVA, and shown as means ± standard deviations. Continuous variables without a normal distribution were examined by the Mann–Whitney U test or Kruskal-Wallis test and presented as median values (interquartile range). Categorical variables were analyzed by the Chi-square test and shown as percentages of participants. The correlation was analyzed by Pearson correlation. In all comparisons, a two-tailed *P*-value < 0.05 was considered statistically significant.

## Results

### Baseline Characteristics of the Study Population

Compared with the control group, females with a history of endometriosis had higher baseline serum E2 levels (**Table 1**, 38.6 *versus* 32.2 pg/mL, *P* = 0.004), lower LH levels (**Table 1**, 4.2 *versus* 4.7 IU/L, *P* = 0.001), lower body mass indexes (BMI, **Table 1**, 20.6 *versus* 21.6 kg/m^2^, *P* = 0.022), and a higher frequency of suffering RIF (**Table 1**, 35.9% *versus* 16.5%, *P* = 0.002). Other baseline data, including maternal age, baseline serum hormone levels (P, FSH, PRL, and T), and percentage of patients in an endometrial preparation program, were similar between the two groups (**Table 1**, *P* > 0.05). The duration of the postoperative period was 3.0 years for the endometriosis group. The baseline characteristics of the subgroups are shown in **Supplementary Table S2**.

**Table 1:** Baseline characteristics of the study population.

	Endometriosis (n = 117)	Control (n = 200)	*P*-value
**Age (years)**	34.0 (32.0, 37.0)	34.0 (31.0, 38.0)	0.912
**BMI (kg/m^2^)**	20.6 (19.1, 22.3)	21.6 (19.5, 23.4)	0.022*
**E2 (pg/mL)**	38.6 (30.7, 53.2)	32.2 (23.9, 46.2)	0.004**
**P (ng/mL)**	0.4 (0.2, 0.5)	0.4 (0.3, 0.6)	0.084
**FSH (IU/L)**	7.3 (5.8, 8.5)	6.9 (5.7, 8.3)	0.430
**LH (IU/L)**	4.2 (3.1, 5.5)	4.7 (3.7, 6.6)	0.001**
**PRL (ng/mL)**	14.8 (12.2, 18.5)	16.2 (11.3, 21.0)	0.276
**T (ng/mL)**	0.2 (0.2, 0.3)	0.2 (0.1, 0.3)	0.982
**Endometrial preparation program**			0.685
Natural cycle	57.3% (67/117)	59.0% (118/200)	
Hormone replacement therapy cycle	42.7% (50/117)	41.0% (82/200)	
**The percentage of RIF/RM/Others**			<0.001***
RIF	35.9% (42/117)	16.5% (33/200)	
RM	17.9% (21/117)	46.0% (92/200)	
Others	46.2% (54/117)	37.5% (75/200)	
**Duration of post-operative period (years)**	3.0 (1.9, 5.5)	/	

BMI, body mass index; E2, estradiol; P, progesterone; FSH, follicle-stimulating hormone; LH, luteinizing hormone; PRL, prolactin; T, testosterone; RIF, repeated implantation failure; RM, recurrent miscarriage; Others, reproductive failure patients who failed to achieve a pregnancy or experienced miscarriage but not meeting the standard of RIF or RM.

Serum hormone levels were all detected on day 3 of the menstrual cycle.

Continuous variables without normal distribution: Mann–Whitney U test, and shown as median (interquartile range); categorical variables: Chi-square test, shown as percentages of participants.

The percentage of RIF: P = 0.002; the percentage of RM: P < 0.001.

*P < 0.05; *P < 0.01; ***P < 0.001.

### Peripheral Immune Responses of Reproductive Failure Patients

Peripheral NK cytotoxicity was significantly decreased in women with a history of endometriosis in comparison with the control group (**Table 2**, 26.6% *versus* 33.3% and 36.1% *versus* 43.3% for cytotoxicity at ET25 and ET50, respectively, both *P* < 0.001). Representative images of NK cytotoxicity for the two groups are shown in **Supplementary Figure S1**. When the endometriosis population was further divided into subgroups according to the location of ectopic lesions (**Table 3**, **Supplementary Figure S2**), NK cytotoxicity in the subgroups of ovarian, pelvic, and mixed endometriosis was still significantly lower than that in the control group (all *P* < 0.05). Interestingly, NK cytotoxicity in women with a history of mixed endometriosis was slightly less than that for other subgroups. Furthermore, peripheral NK cytotoxicity was significantly decreased in the endometriosis group from that of the control group in the “Other” population (**Supplementary Tables S3, 4**). The same tendency was observed in the RIF and RM population, but without statistical significance (**Supplementary Tables S5–8**). Regarding the mechanisms of low PBMC cytotoxicity in patients with endometriosis, the toxic granules (granzyme B, perforin, and granulysin) and surface receptors (NKG2D, NKp30, NKp46, CD158a, and CD158b) of peripheral NK cells were analyzed. Nevertheless, all parameters were comparable between the control and endometriosis groups (all *P* > 0.05, **Supplementary Figure S3**). Negative results were also observed when analyzing the productions of IFN-γ and TNF-α by CD4^+^ T cells in the endometriosis group compared with the control group (**Supplementary Figure S4**). More research should be conducted to determine the underlying mechanisms. The percentages of peripheral lymphocytes subsets were comparable among the groups (**Tables 2**, **3**, **Supplementary Figure S1**, *P* > 0.05).

**Table 2:** Comparison of lymphocyte subsets from endometrium or peripheral blood and cytotoxicity of peripheral NK cells between females with or without a history of endometriosis.

	Endometriosis (n = 117)	Control (n = 200)	*P*-value
**eCD56^+^ (%)**	11.7 (7.9, 19.3)	12.1 (8.5, 19.1)	0.607
**eCD68^+^ (%)**	2.3 (1.6, 3.0)	2.1 (1.5, 2.7)	0.167
**eCD1a^+^ (%)**	0.06 (0.04, 0.10)	0.05 (0.03, 0.10)	0.183
**eCD83^+^ (%)**	1.8 (1.3, 2.4)	1.7 (1.2, 2.5)	0.760
**eCD57^+^ (%)**	0.4 (0.2, 0.5)	0.3 (0.2, 0.5)	0.558
**eCD8^+^ (%)**	2.9 (2.1, 4.0)	3.0 (1.9, 4.2)	0.684
**eFoxP3^+^ (%)**	0.10 (0.06, 0.14)	0.09 (0.06, 0.13)	0.512
**pCD3^+^ (%)**	69.4 ± 6.9	68.4 ± 8.2	0.288
**pCD3^+^CD8^+^ (%)**	26.1 (22.5, 30.8)	25.6 (22.5, 29.8)	0.931
**pCD3^+^CD4^+^ (%)**	37.1 ± 6.4	35.8 ± 6.7	0.094
**pCD56^+^CD16^+^ (%)**	15.3 (11.6, 20.3)	16.9 (11.1, 21.7)	0.564
**pCD19^+^ (%)**	12.1 (9.9, 15.7)	12.7 (10.5, 15.1)	0.775
**Cytotoxicity at ET25 (%)**	26.6 ± 14.5	33.3 ± 14.5	<0.001***
**Cytotoxicity at ET50 (%)**	36.1 ± 15.6	43.3 ± 15.2	<0.001***

e, endometrial cell; p, peripheral blood mononuclear cell.

Cytotoxicity, cytotoxicity of peripheral NK cells, shown as the ratios of target cell lysis. ET, the ratios between effectors and target cells.

Continuous variables with normal distribution: t-test, and shown as mean ± standard deviation; Continuous variables without normal distribution: Mann–Whitney U test, and shown as median (interquartile range).

***P < 0.001.

**Table 3:** Comparison of lymphocyte subsets from endometrium or peripheral blood and cytotoxicity of peripheral NK cells among females in different endometriosis subgroups and the control group.

	Ovarian endometriosis (n = 44)	Pelvic endometriosis (n = 57)	Mixed endometriosis (n = 16)	Control (n = 200)	*P*-value
**eCD56^+^ (%)**	11.8 (8.5, 20.8)	12.0 (7.3, 19.8)	10.6 (8.1, 16.1)	12.1 (8.5, 19.1)	0.893
**eCD68^+^ (%)**	2.0 (1.3, 2.7)	2.3 (1.6, 3.3)	2.8 (2.3, 3.1)	2.1 (1.5, 2.7)	0.035*
**eCD1a^+^ (%)**	0.06 (0.04, 0.09)	0.07 (0.04, 0.11)	0.06 (0.03, 0.07)	0.05 (0.03, 0.10)	0.424
**eCD83^+^ (%)**	1.6 (1.3, 2.2)	1.9 (1.1, 2.4)	2.6 (1.6, 4.1)	1.7 (1.2, 2.5)	0.304
**eCD57^+^ (%)**	0.4 (0.2, 0.5)	0.4 (0.3, 0.6)	0.3 (0.2, 0.4)	0.3 (0.2, 0.5)	0.207
**eCD8^+^ (%)**	2.7 (1.7, 3.9)	3.1 (2.3, 4.1)	2.6 (2.1, 3.9)	3.0 (1.9, 4.2)	0.626
**eFoxP3^+^ (%)**	0.08 (0.05, 0.11)	0.11 (0.07, 0.15)	0.08 (0.06, 0.17)	0.09 (0.06, 0.13)	0.082
**pCD3^+^ (%)**	69.1 ± 7.2	69.5 ± 6.4	70.2 ± 7.8	68.4 ± 8.2	0.709
**pCD3^+^CD8^+^ (%)**	26.0 (22.3, 32.0)	25.7 (23.1, 30.6)	26.7 (22.0, 30.8)	25.6 (22.5, 29.8)	0.984
**pCD3^+^CD4^+^ (%)**	36.8 ± 6.0	37.2 ± 6.6	37.3 ± 7.3	35.8 ± 6.7	0.403
**pCD56^+^CD16^+^ (%)**	15.9 (9.8, 20.4)	15.3 (11.9, 21.1)	15.2 (11.2, 19.8)	16.9 (11.1, 21.7)	0.951
**pCD19^+^ (%)**	11.7 (10.4, 15.8)	13.2 (10.2, 15.0)	11.8 (8.4, 14.2)	12.7 (10.5, 15.1)	0.620
**Cytotoxicity at ET25 (%)**	27.6 ± 16.1	27.6 ± 14.0	20.2 ± 9.9	33.3 ± 14.5	<0.001***
**Cytotoxicity at ET50 (%)**	36.7 ± 17.3	37.4 ± 14.5	29.6 ± 13.1	43.4 ± 15.2	<0.001***

e, endometrial cell; p, peripheral blood mononuclear cell; Mixed endometriosis, endometriotic lesions within both the pelvis and ovary.

Cytotoxicity, cytotoxicity of peripheral NK cells, shown as the ratios of target cell lysis. ET, the ratios between effectors and target cells.

Continuous variables with normal distribution: ANOVA, and shown as mean ± standard deviation; continuous variables without normal distribution: Kruskal-Wallis test, and shown as median (interquartile range).

eCD68: Mixed endometriosis versus Control, P = 0.043.

Cytotoxicity at ET25: Ovarian endometriosis versus Control, P = 0.020; Pelvic endometriosis versus Control, P = 0.009; Mixed endometriosis versus Control, P = 0.001.

Cytotoxicity at ET50: Ovarian endometriosis versus Control, P = 0.009; Pelvic endometriosis versus Control, P = 0.010; Mixed endometriosis versus Control, P = 0.001.

*P < 0.05; ***P < 0.001.

### Endometrial Immune Cell Profiles of Reproductive Failure Patients

**Figure 1:**
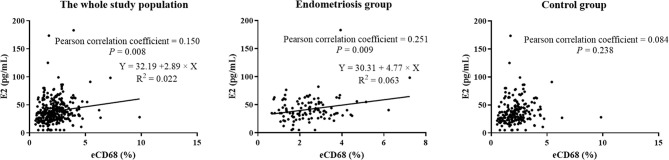
Weak correlation between E2 and endometrial CD68 in the study population.

There was no statistically significant difference between women with a history of endometriosis and the control group regarding endometrial immunological parameters (**Table 2**, *P* > 0.05). Representative images of the uterine immune cells of patients are shown in **Supplementary Figure S5**. However, when the endometriosis group was further divided into subgroups (**Table 3**, **Supplementary Figure S6**), the proportion of endometrial CD68^+^ macrophages in women with mixed endometriosis was significantly higher than that in the control group (2.8% *versus* 2.1%, *P* = 0.043), indicating that uterine macrophages are associated with mixed endometriosis. The remaining uterine parameters were still comparable among the subgroups (**Table 3**, *P* > 0.05). Intriguingly, there was a weakly positive correlation between the proportion of endometrial CD68^+^ macrophages and baseline serum E2 levels, indicating a potential association among E2, local immune profiles, and endometriosis (**Figure 1**, **Supplementary Table S9**, r = 0.251, *P* = 0.009). EndometDB data also showed that CD68 was slightly positively correlated with ESR2, but not with ESR1 (**Supplementary Figure S7**).

## Discussion

The hormone levels, BMI, peripheral NK cytotoxicity, and endometrial immune cell subtypes of the endometriosis population were significantly different from those of the control group. In this study, women with a history of endometriosis, especially those exhibiting overlap of multiple diseases (mixed endometriosis), were associated with decreased NK cytotoxicity and changed local immune cell profiles. At present, the most commonly used disease staging system (the R-ASRM classification) is based on the number and depth of lesions, endometrioma, and adhesions within the pelvic cavity (20). However, only a marginal relationship was demonstrated among the number of lesions, lesion symptoms, the severity of the disease, and the overall impact on quality of life (except for an association between pelvic pain and deep infiltrating endometriosis sites) (3). Thus, overlap of multiple diseases might provide a new disease staging system for endometriosis. Additionally, a slightly positive correlation between baseline E2 levels and endometrial CD68^+^ macrophages in women with a history of endometriosis indicated a potential association among hormones, immune cell responses, and endometriosis.

Patients with a history of endometriosis had a higher prevalence of RIF, whose main etiology is low endometrial receptivity. Regarding the hormone effects, continual high expression of estrogen in women with a history of endometriosis likely interferes with the transition from proliferative to secretory phases and other important reproductive events (21). According to an analysis of immune responses, significantly increased endometrial CD68^+^ macrophages were observed in women with a history of mixed endometriosis compared to the control group. Macrophage-secreted proinflammatory cytokines, such as tumor necrosis factor-α, IL-15, and macrophage inflammatory protein 1B, could influence preparation of the endometrium for implantation (22). Macrophages also regulate the vascular network *via* the production of cytokines (such as IL-6, IL-10, IL-12, and TGF-β1) (23, 24), which further regulates endometrial receptivity. Furthermore, endometriotic lesions secreting both inflammatory cytokines and reactive oxygen species could lead to the production of DNA and cytoplasmic fragmentation in embryos (25). This would also result in RIF.

There was a significant relationship between a history of endometriosis and decreased peripheral NK cytotoxicity in this study. Some studies also suggested that NK cytotoxicity in peripheral blood and peritoneal fluid was decreased in endometriosis patients compared to controls (26–28). A review by Sciezynska et al. (29) showed that factors including IL-6, IL-10, IL-12, IL-15, and TGF-β were involved in the inhibition of NK cell cytotoxicity during the course of endometriosis. For example, the interaction between macrophages and ectopic endometrial stromal cells could downregulate the expression of NKG2D, perforin, and IFN-γ, as well as the cytotoxicity of NK cells by the secretion of IL-10 and TGF-β (30). IL-6 or peritoneal fluid from endometriosis patients reduces the cytolytic activity of NK cells, concomitantly with the down-regulation of granzyme B, perforin, and the killer activating receptor (NKp46), while increasing the killer inhibitory receptor (CD158b) (31). Moreover, platelet-derived TGF-β1 suppresses the expression of killer activating receptor (NKG2D) on peritoneal NK cells, resulting in reduced cytotoxicity in women with endometriosis; however, neutralization of TGF-β1 reversed this reduction (32). Increased expression of killer inhibitory receptor (CD158a) by circulating NK cells has also been observed in patients with endometriosis (33). However, differential expression of the toxic granules (granzyme B, perforin, and granulysin) and surface receptors (NKG2D, NKp30, NKp46, CD158a, and CD158b) of peripheral NK cells between females with or without endometriosis was not observed in our study (**Supplementary Figure S3**). Circulating NK cytotoxicity might be regulated by other factors, such as other surface receptors (NKp44, NKG2A, ITAM-KIR, etc.), the production of IFN-γ and TNF-α, NK cell maturation and development, proangiogenic features of NK cells, or the interaction with other immune cells such as regulatory T cells (34–38). It is worth noting that, although PBMC cytotoxicity is used to represent NK cytotoxicity in many studies, peripheral T cells might also exhibit cytotoxicity *via* the production of cytokines, such as IFN-γ, TNF-α, and the human cytotoxic factor (39, 40). However, our data showed that the production of IFN-γ and TNF-α by CD4^+^ T cells in the endometriosis group were comparable with that in the control group (**Supplementary Figure S4**). Therefore, further study is required, such as single-cell sequencing of peripheral lymphocytes, to verify the underlying mechanism and detect the function of each immune cell type. Nevertheless, it should be stressed that some studies could not confirm abrogated circulating NK cytotoxicity against K562 (41, 42). For example, Ho et al. (42) found that women with endometriosis showed a decrease in peritoneal NK cytotoxicity against K562; however, the results of circulating NK cytotoxicities were comparable between endometriosis and non-endometriosis groups. Furthermore, a study by Vigano et al. (41) showed decreased cytotoxicity of PBMC against endometrial stromal cells but not K562. Some of these negative results might be explained by the low number of patients and controls and population differences.

Endometriosis was correlated with local macrophages. There is also some evidence that the macrophage proportion is enhanced in the peritoneal fluid and endometrium of women with endometriosis (43, 44). In the endometriotic microenvironment, macrophages are a major source of elevated proinflammatory and chemotactic cytokines and angiogenic mediators (45), promoting the proliferation and invasion of endometrial stromal cells. Macrophages also produce anti-inflammatory cytokines, and play a critical role in accelerating the growth, adhesion, invasion, epithelial-mesenchymal transition, fibrogenesis, and angiogenesis of ectopic lesions, as well as the impairment of immune surveillance (24). Additionally, macrophages may acquire memory-like characteristics to regulate endometriosis in low inflammatory microenvironments (46). Enhanced macrophages in the endometriotic microenvironment could promote the proliferation and invasion of endometrial stromal cells, and enhance the growth, adhesion, and invasion of ectopic lesions, which might lead to the appearance of ectopic lesions in multiple locations. On the other hand, the endometriotic microenvironment could recruit macrophages (47, 48). Cause and effect are hard to distinguish, indicating the need for further research to determine the upstream and downstream mechanisms of macrophages in endometriosis. However, Braun et al. (49) showed a decrease of macrophages in the same endometrial phase in patients with endometriosis, which might be due to the small sample size or different population. Furthermore, it is worth noting that the *P*-value (0.043) of increased macrophages in the mixed endometriosis group was very close to 0.05; thus, this should be analyzed further with a larger or validation dataset. The proportions of CD56^+^ NK cells did not change in this study, which may be due to the fact that their functions in endometriosis depend on their cytotoxicity and not on cell numbers.

A history of endometriosis was correlated with decreased BMI. A consistent inverse association has also been observed between BMI and endometriosis in other studies (50, 51), which may also relate to hormonal differences between heavier and lighter women. Moreover, obesity is associated with lower E2 levels (52, 53). In this study, a history of endometriosis was also associated with increased baseline E2 levels, with a weak correlation between baseline E2 levels and uterine macrophages. Previous work has shown that that macrophage infiltration of endometriosis increases in response to E2 (47). Estrogen-stimulated uterine growth factors such as the colony-stimulating factor-1 or granulocyte-macrophage colony-stimulating factor promote macrophage migration and infiltration (48). These partly explain why E2 and macrophages were associated with endometriosis. Due to the difference of sampling dates, this study further analyzed the correlation between CD68 and estrogen receptors in EndometDB. The data in EndometDB showed that CD68 was slightly positively correlated with ESR2, but not ESR1 (**Supplementary Figure S7**). Previous research has shown that ESR2 upregulates CCL2 *via* NF-κB signaling in endometriotic stromal cells and recruits macrophages to promote the pathogenesis of endometriosis (54). On the other hand, M1 and M2 macrophage infiltration is comparable between ESR1 knockout mice and wild type mice (55), which suggests the potential effect of ESR2 in the recruitment of macrophages. This, therefore, suggests the possibility of developing appropriate antagonists of macrophage estrogen signaling as novel therapeutic agents in endometriosis.

Nowadays, the gold standard for diagnosis of endometriosis is still the invasive laparoscopic visualization of lesions (56). The main treatments for this disease include hormone therapies, with limited effectiveness and considerable side effects, and invasive surgery (3). The recurrence rate after surgery is high, and repeat surgery increases the risk of premature ovarian failure, adhesion, and organ injury (57). Therefore, the findings of this study regarding immune responses, and the interaction between endocrine and immune responses in women with endometriosis, provide new insights for the diagnosis and treatment of endometriosis. Although this study demonstrated an association between immune responses and a history of endometriosis, it still has limitations. Firstly, it is a retrospective study; thus, a well-designed prospective study should be conducted to verify the above results and determine more potential mechanisms. The expression of surface receptors, the secretion of cytotoxic granules and cytokines of peripheral NK, and the cytokine secretion of peripheral T cells should also be collected from the entire study population. Subgroups of macrophages, NK cells, T cells, and dendritic cells should also be analyzed in future research. Secondly, basic research should be performed to verify the underlying mechanisms of low peripheral lymphocyte cytotoxicity, and to study the immune cell profiles of ectopic and eutopic endometria, as well as the functions and mechanisms of each immune cell type. Finally, not all patients underwent laparoscopic observations to confirm the absence of endometriosis in the control and endometriosis group at the time of endometrial and peripheral blood sampling. Moreover, there were no totally healthy control patients in this study. Therefore, future work should pay close attention to the inclusion and exclusion criteria in order to limit the unknown bias.

## Data Availability Statement

The original contributions presented in the study are included in the article/**Supplementary Material**. Further inquiries can be directed to the corresponding authors.

## Ethics Statement

The studies involving human participants were reviewed and approved by The ethics committee of Shenzhen Zhongshan Urology Hospital. The patients/participants provided their written informed consent to participate in this study.

## Author Contributions

LW and LL: Project development, Data management, Manuscript writing/editing. YL, CH and RL: Interpretation, Manuscript writing. TW, JM and YaZ: Data collection, Data evaluation. YC: Help for the revision of the manuscript. LD and YoZ: Protocol/project development, Interpretation, Management. All authors contributed to the article and approved the submitted version.

## Funding

The project was funded by National Key Research & Developmental Program of China (2018YFC1003900, 2018YFC1003904), Guangdong Basic and Applied Basic Research Foundation (2019A1515011315), Basic Research Program of Shenzhen (JCYJ20190813161010761), National Natural Science Foundation of China (No. 81701529), and Sanming Project of Medicine in Shenzhen (SZSM201502035).

## Conflict of Interest

The authors declare that the research was conducted in the absence of any commercial or financial relationships that could be construed as a potential conflict of interest.

## Publisher’s Note

All claims expressed in this article are solely those of the authors and do not necessarily represent those of their affiliated organizations, or those of the publisher, the editors and the reviewers. Any product that may be evaluated in this article, or claim that may be made by its manufacturer, is not guaranteed or endorsed by the publisher.

## References

[1] Zondervan KT Becker CM Missmer SA. Endometriosis. N Engl J Med (2020) 382(13):1244–56. doi: 10.1056/NEJMra1810764 32212520

[2] Garcia-Penarrubia P Ruiz-Alcaraz AJ Martinez-Esparza M Marin P Machado-Linde F. Hypothetical Roadmap Towards Endometriosis: Prenatal Endocrine-Disrupting Chemical Pollutant Exposure, Anogenital Distance, Gut-Genital Microbiota and Subclinical Infections. Hum Reprod Updat (2020) 26(2):214–46. doi: 10.1093/humupd/dmz044 32108227

[3] As-Sanie S Black R Giudice LC Gray Valbrun T Gupta J Jones B. Assessing Research Gaps and Unmet Needs in Endometriosis. Am J Obstet Gynecol (2019) 221(2):86–94. doi: 10.1016/j.ajog.2019.02.033 30790565

[4] Uccella S Manzoni P Cromi A Marconi N Gisone B Miraglia A. Pregnancy After Endometriosis: Maternal and Neonatal Outcomes According to the Location of the Disease. Am J Perinatol (2019) 36(S 02):S91–S8. doi: 10.1055/s-0039-1692130 31238367

[5] Vercellini P Vigano P Somigliana E Fedele L. Endometriosis: Pathogenesis and Treatment. Nat Rev Endocrinol (2014) 10(5):261–75. doi: 10.1038/nrendo.2013.255 24366116

[6] Vallve-Juanico J Houshdaran S Giudice LC. The Endometrial Immune Environment of Women With Endometriosis. Hum Reprod Updat (2019) 25(5):564–91. doi: 10.1093/humupd/dmz018 PMC6737540 31424502

[7] Zondervan KT Becker CM Koga K Missmer SA Taylor RN Vigano P. Endometriosis. Nat Rev Dis Primers (2018) 4(1):9. doi: 10.1038/s41572-018-0008-5 30026507

[8] Hudson QJ Ashjaei K Perricos A Kuessel L Husslein H Wenzl R. Endometriosis Patients Show an Increased M2 Response in the Peritoneal CD14(+low)/CD68(+low) Macrophage Subpopulation Coupled With an Increase in the T-Helper 2 and T-Regulatory Cells. Reprod Sci (2020) 27(10):1920–31. doi: 10.1007/s43032-020-00211-9 PMC7452931 32572831

[9] Fukui A Mai C Saeki S Yamamoto M Takeyama R Kato T. Pelvic Endometriosis and Natural Killer Cell Immunity. Am J Reprod Immunol (2021) 85(4):e13342. doi: 10.1111/aji.13342 32896016

[10] Tariverdian N Siedentopf F Rucke M Blois SM Klapp BF Kentenich H. Intraperitoneal Immune Cell Status in Infertile Women With and Without Endometriosis. J Reprod Immunol (2009) 80(1-2):80–90. doi: 10.1016/j.jri.2008.12.005 19375804

[11] Schulke L Berbic M Manconi F Tokushige N Markham R Fraser IS. Dendritic Cell Populations in the Eutopic and Ectopic Endometrium of Women With Endometriosis. Hum Reprod (2009) 24(7):1695–703. doi: 10.1093/humrep/dep071 19321495

[12] Izumi G Koga K Takamura M Makabe T Nagai M Urata Y. Mannose Receptor is Highly Expressed by Peritoneal Dendritic Cells in Endometriosis. Fertil Steril (2017) 107(1):167–73 e2. doi: 10.1016/j.fertnstert.2016.09.036 27793384

[13] Tanaka Y Mori T Ito F Koshiba A Takaoka O Kataoka H. Exacerbation of Endometriosis Due To Regulatory T-Cell Dysfunction. J Clin Endocrinol Metab (2017) 102(9):3206–17. doi: 10.1210/jc.2017-00052 28575420

[14] Khan KN Yamamoto K Fujishita A Muto H Koshiba A Kuroboshi H. Differential Levels of Regulatory T Cells and T-Helper-17 Cells in Women With Early and Advanced Endometriosis. J Clin Endocrinol Metab (2019) 104(10):4715–29. doi: 10.1210/jc.2019-00350 31042291

[15] Guo M Bafligil C Tapmeier T Hubbard C Manek S Shang C. Mass Cytometry Analysis Reveals a Distinct Immune Environment in Peritoneal Fluid in Endometriosis: A Characterisation Study. BMC Med (2020) 18(1):3. doi: 10.1186/s12916-019-1470-y 31907005 PMC6945609

[16] Riccio LGC Baracat EC Chapron C Batteux F Abrao MS. The Role of the B Lymphocytes in Endometriosis: A Systematic Review. J Reprod Immunol (2017) 123:29–34. doi: 10.1016/j.jri.2017.09.001 28910679

[17] Wan CY Song C Diao LH Li GG Bao ZJ Hu XD. Laser-Assisted Hatching Improves Clinical Outcomes of Vitrified-Warmed Blastocysts Developed From Low-Grade Cleavage-Stage Embryos: A Prospective Randomized Study. Reprod BioMed Online (2014) 28(5):582–9. doi: 10.1016/j.rbmo.2014.01.006 24631166

[18] Li Y Yu S Huang C Lian R Chen C Liu S. Evaluation of Peripheral and Uterine Immune Status of Chronic Endometritis in Patients With Recurrent Reproductive Failure. Fertil Steril (2020) 113(1):187–96 e1. doi: 10.1016/j.fertnstert.2019.09.001 31718829

[19] Gabriel M Fey V Heinosalo T Adhikari P Rytkonen K Komulainen T. A Relational Database to Identify Differentially Expressed Genes in the Endometrium and Endometriosis Lesions. Sci Data (2020) 7(1):284. doi: 10.1038/s41597-020-00623-x 32859947 PMC7455745

[20] Keckstein J Hudelist G. Classification of Deep Endometriosis (DE) Including Bowel Endometriosis: From R-ASRM to #Enzian-Classification. Best Pract Res Clin Obstet Gynaecol (2021) 71:27–37. doi: 10.1016/j.bpobgyn.2020.11.004 33558167

[21] Fox C Morin S Jeong JW Scott RT Jr. Lessey BA. Local and Systemic Factors and Implantation: What is the Evidence? Fertil Steril (2016) 105(4):873–84. doi: 10.1016/j.fertnstert.2016.02.018 PMC4821679 26945096

[22] Gnainsky Y Granot I Aldo PB Barash A Or Y Schechtman E. Local Injury of the Endometrium Induces an Inflammatory Response That Promotes Successful Implantation. Fertil Steril (2010) 94(6):2030–6. doi: 10.1016/j.fertnstert.2010.02.022 PMC3025806 20338560

[23] Hanada T Tsuji S Nakayama M Wakinoue S Kasahara K Kimura F. Suppressive Regulatory T Cells and Latent Transforming Growth Factor-Beta-Expressing Macrophages are Altered in the Peritoneal Fluid of Patients With Endometriosis. Reprod Biol Endocrinol (2018) 16(1):9. doi: 10.1186/s12958-018-0325-2 29391020 PMC5796574

[24] Zhou WJ Yang HL Shao J Mei J Chang KK Zhu R. Anti-Inflammatory Cytokines in Endometriosis. Cell Mol Life Sci (2019) 76(11):2111–32. doi: 10.1007/s00018-019-03056-x PMC11105498 30826860

[25] Stilley JA Birt JA Sharpe-Timms KL. Cellular and Molecular Basis for Endometriosis-Associated Infertility. Cell Tissue Res (2012) 349(3):849–62. doi: 10.1007/s00441-011-1309-0 PMC3429772 22298022

[26] Wong KH Simon JA. *In Vitro* Effect of Gonadotropin-Releasing Hormone Agonist on Natural Killer Cell Cytolysis in Women With and Without Endometriosis. Am J Obstet Gynecol (2004) 190(1):44–9. doi: 10.1016/j.ajog.2003.08.032 14749633

[27] Wu MY Yang JH Chao KH Hwang JL Yang YS Ho HN. Increase in the Expression of Killer Cell Inhibitory Receptors on Peritoneal Natural Killer Cells in Women With Endometriosis. Fertil Steril (2000) 74(6):1187–91. doi: 10.1016/s0015-0282(00)01592-2 11119748

[28] Tanaka E Sendo F Kawagoe S Hiroi M. Decreased Natural Killer Cell Activity in Women With Endometriosis. Gynecol Obstet Invest (1992) 34(1):27–30. doi: 10.1159/000292720 1526528

[29] Sciezynska A Komorowski M Soszynska M Malejczyk J. NK Cells as Potential Targets for Immunotherapy in Endometriosis. J Clin Med (2019) 8(9):1468. doi: 10.3390/jcm8091468 31540116 PMC6780982

[30] Yang HL Zhou WJ Chang KK Mei J Huang LQ Wang MY. The Crosstalk Between Endometrial Stromal Cells and Macrophages Impairs Cytotoxicity of NK Cells in Endometriosis by Secreting IL-10 and TGF-Beta. Reproduction (2017) 154(6):815–25. doi: 10.1530/REP-17-0342 28971893

[31] Kang YJ Jeung IC Park A Park YJ Jung H Kim TD. An Increased Level of IL-6 Suppresses NK Cell Activity in Peritoneal Fluid of Patients With Endometriosis *via* Regulation of SHP-2 Expression. Hum Reprod (2014) 29(10):2176–89. doi: 10.1093/humrep/deu172 25035432

[32] Guo SW Du Y Liu X. Platelet-Derived TGF-Beta1 Mediates the Down-Modulation of NKG2D Expression and may be Responsible for Impaired Natural Killer (NK) Cytotoxicity in Women With Endometriosis. Hum Reprod (2016) 31(7):1462–74. doi: 10.1093/humrep/dew057 27130956

[33] Zhang C Maeda N Izumiya C Yamamoto Y Kusume T Oguri H. Killer Immunoglobulin-Like Receptor and Human Leukocyte Antigen Expression as Immunodiagnostic Parameters for Pelvic Endometriosis. Am J Reprod Immunol (2006) 55(2):106–14. doi: 10.1111/j.1600-0897.2005.00332.x 16433829

[34] Thiruchelvam U Wingfield M O’Farrelly C. Natural Killer Cells: Key Players in Endometriosis. Am J Reprod Immunol (2015) 74(4):291–301. doi: 10.1111/aji.12408 26104509

[35] Yuan D Koh CY Wilder JA. Interactions Between B Lymphocytes and NK Cells. FASEB J (1994) 8(13):1012–8. doi: 10.1096/fasebj.8.13.7926365 7926365

[36] Zhou J Hu M Li J Liu Y Luo J Zhang L. Mannan-Binding Lectin Regulates Inflammatory Cytokine Production, Proliferation, and Cytotoxicity of Human Peripheral Natural Killer Cells. Mediators Inflamm (2019) 2019:6738286. doi: 10.1155/2019/6738286 31915415 PMC6930792

[37] Radomska-Lesniewska DM Bialoszewska A Kaminski P. Angiogenic Properties of NK Cells in Cancer and Other Angiogenesis-Dependent Diseases. Cells (2021) 10(7):1621. doi: 10.3390/cells10071621 34209508 PMC8303392

[38] Vourc’h M David G Gaborit B Broquet A Jacqueline C Chaumette T. Pseudomonas Aeruginosa Infection Impairs NKG2D-Dependent NK Cell Cytotoxicity Through Regulatory T-Cell Activation. Infect Immun (2020) 88(12):e00363–20. doi: 10.1128/IAI.00363-20 PMC7671885 32928966

[39] Iwahori K Shintani Y Funaki S Yamamoto Y Matsumoto M Yoshida T. Peripheral T Cell Cytotoxicity Predicts T Cell Function in the Tumor Microenvironment. Sci Rep (2019) 9(1):2636. doi: 10.1038/s41598-019-39345-5 30796310 PMC6385254

[40] Agarwal R Chaturvedi UC Misra A Mukerjee R Kapoor S Nagar R. Production of Cytotoxic Factor by Peripheral Blood Mononuclear Cells (PBMC) in Patients With Dengue Haemorrhagic Fever. Clin Exp Immunol (1998) 112(3):477–81. doi: 10.1046/j.1365-2249.1998.00598.x PMC1904990 9649218

[41] Vigano P Vercellini P Di Blasio AM Colombo A Candiani GB Vignali M. Deficient Antiendometrium Lymphocyte-Mediated Cytotoxicity in Patients With Endometriosis. Fertil Steril (1991) 56(5):894–9. doi: 10.1016/s0015-0282(16)54661-5 1936324

[42] Ho HN Chao KH Chen HF Wu MY Yang YS Lee TY. Peritoneal Natural Killer Cytotoxicity and CD25+ CD3+ Lymphocyte Subpopulation are Decreased in Women With Stage III-IV Endometriosis. Hum Reprod (1995) 10(10):2671–5. doi: 10.1093/oxfordjournals.humrep.a135765 8567790

[43] Beste MT Pfaffle-Doyle N Prentice EA Morris SN Lauffenburger DA Isaacson KB. Molecular Network Analysis of Endometriosis Reveals a Role for C-Jun-Regulated Macrophage Activation. Sci Transl Med (2014) 6(222):222ra16. doi: 10.1126/scitranslmed.3007988 PMC4118592 24500404

[44] Forster R Sarginson A Velichkova A Hogg C Dorning A Horne AW. Macrophage-Derived Insulin-Like Growth Factor-1 is a Key Neurotrophic and Nerve-Sensitizing Factor in Pain Associated With Endometriosis. FASEB J (2019) 33(10):11210–22. doi: 10.1096/fj.201900797R PMC6766660 31291762

[45] Sekulovski N Whorton AE Tanaka T Hirota Y Shi M MacLean JA. Niclosamide Suppresses Macrophage-Induced Inflammation in Endometriosisdagger. Biol Reprod (2020) 102(5):1011–9. doi: 10.1093/biolre/ioaa010 PMC7186788 31950153

[46] Jeljeli M Riccio LGC Chouzenoux S Moresi F Toullec L Doridot L. Macrophage Immune Memory Controls Endometriosis in Mice and Humans. Cell Rep (2020) 33(5):108325. doi: 10.1016/j.celrep.2020.108325 33147452

[47] Yilmaz BD Bulun SE. Endometriosis and Nuclear Receptors. Hum Reprod Updat (2019) 25(4):473–85. doi: 10.1093/humupd/dmz005 PMC6601390 30809650

[48] Greaves E Temp J Esnal-Zufiurre A Mechsner S Horne AW Saunders PT. Estradiol is a Critical Mediator of Macrophage-Nerve Cross Talk in Peritoneal Endometriosis. Am J Pathol (2015) 185(8):2286–97. doi: 10.1016/j.ajpath.2015.04.012 PMC4530129 26073038

[49] Braun DP Ding J Shen J Rana N Fernandez BB Dmowski WP. Relationship Between Apoptosis and the Number of Macrophages in Eutopic Endometrium From Women With and Without Endometriosis. Fertil Steril (2002) 78(4):830–5. doi: 10.1016/s0015-0282(02)03334-4 12372464

[50] Hager M Wenzl R Riesenhuber S Marschalek J Kuessel L Mayrhofer D. The Prevalence of Incidental Endometriosis in Women Undergoing Laparoscopic Ovarian Drilling for Clomiphene-Resistant Polycystic Ovary Syndrome: A Retrospective Cohort Study and Meta-Analysis. J Clin Med (2019) 8(8):1210. doi: 10.3390/jcm8081210 31416144 PMC6722764

[51] Farland LV Degnan WJ Harris HR Tobias DK Missmer SA. A Prospective Study of Endometriosis and Risk of Type 2 Diabetes. Diabetologia (2021) 64(3):552–60. doi: 10.1007/s00125-020-05347-6 PMC8609862 33399910

[52] Stanikova D Luck T Pabst A Bae YJ Hinz A Glaesmer H. Associations Between Anxiety, Body Mass Index, and Sex Hormones in Women. Front Psychiatry (2019) 10:479. doi: 10.3389/fpsyt.2019.00479 31333520 PMC6620895

[53] Hampton T. How Targeting Fat Cells’ Estrogen Receptors Could Fight Obesity. JAMA (2020) 324(21):2146. doi: 10.1001/jama.2020.22148 33258874

[54] Gou Y Li X Li P Zhang H Xu T Wang H. Estrogen Receptor Beta Upregulates CCL2 *via* NF-kappaB Signaling in Endometriotic Stromal Cells and Recruits Macrophages to Promote the Pathogenesis of Endometriosis. Hum Reprod (2019) 34(4):646–58. doi: 10.1093/humrep/dez019 30838396

[55] Burns KA Thomas SY Hamilton KJ Young SL Cook DN Korach KS. Early Endometriosis in Females Is Directed by Immune-Mediated Estrogen Receptor Alpha and IL-6 Cross-Talk. Endocrinology (2018) 159(1):103–18. doi: 10.1210/en.2017-00562 PMC5761597 28927243

[56] Vanhie A O D Peterse D Beckers A Cuellar A Fassbender A. Plasma miRNAs as Biomarkers for Endometriosis. Hum Reprod (2019) 34(9):1650–60. doi: 10.1093/humrep/dez116 PMC6736379 31411334

[57] Guo SW Martin DC. The Perioperative Period: A Critical Yet Neglected Time Window for Reducing the Recurrence Risk of Endometriosis? Hum Reprod (2019) 34(10):1858–65. doi: 10.1093/humrep/dez187 31585460

