# Supplementary material for: A History of Endometriosis Is Associated With Decreased Peripheral NK Cytotoxicity and Increased Infiltration of Uterine CD68+ Macrophages
Source: Front Immunol. 2021 Aug 31;12:711231. doi: 10.3389/fimmu.2021.711231 (PMC8438297; doi:10.3389/fimmu.2021.711231)
Supplement: Supplementary file 1 [file DataSheet_1.docx]

Supplementary Material

# Supplementary Tables

**Supplementary Table S1** Summary of primary antibodies and immunohistochemical technique.

| **Antibodies** | **Source** | **Clone** | **Dilution** | **Antigen retrieval** |
| --- | --- | --- | --- | --- |
| CD56 | Gene | 12303 | 1 : 200 | Citrate acid |
| CD68 | Novcastra | KP1 | 1 : 150 | EDTA |
| CD1a | Novcastra | MTB1 | 1 : 30 | EDTA |
| CD83 | Sigma-Aldrich | HB15e | 1 : 150 | EDTA |
| CD57 | Thermo | NK1 | 1 : 100 | Citrate acid |
| CD8 | Novcastra | 4B11 | 1 : 150 | Citrate acid |
| FoxP3 | eBioscience | 236A/E7 | 1 : 100 | EDTA |

Note: Citrate acid: PH = 5.80-6.00, EDTA (ethylenediaminetetraacetic acid): PH = 8.95-9.05.

**Supplementary Table S2** Baseline characteristics in the subgroups.

|  | **Ovarian endometriosis (n = 44)** | **Pelvic endometriosis (n = 57)** | **Mixed endometriosis (n = 16)** | **Control (n = 200)** | ***P*-value** |
| --- | --- | --- | --- | --- | --- |
| **Age (years)** | 34.0 (31.0, 36.0) | 35.0 (32.0, 38.0) | 34.0 (30.3, 38.0) | 34.0 (31.0, 38.0) | 0.286 |
| **BMI (kg/m^2^)** | 20.3 (18.9, 22.2) | 20.8 (19.2, 23.0) | 20.2 (18.4, 21.8) | 21.6 (19.5, 23.4) | 0.080 |
| **E2 (pg/mL)** | 44.5 (34.2, 56.7) | 35.0 (25.8, 44.2) | 37.9 (28.0, 52.0) | 32.2 (23.9, 46.2) | **0.005**** |
| **P (ng/mL)** | 0.4 (0.2, 0.5) | 0.4 (0.3, 0.5) | 0.2 (0.1, 0.6) | 0.4 (0.3, 0.6) | 0.225 |
| **FSH (IU/L)** | 7.3 (5.5, 9.0) | 6.9 (6.0, 7.9) | 7.5 (5.9, 8.5) | 6.9 (5.7, 8.3) | 0.808 |
| **LH (IU/L)** | 4.1 (2.6, 5.4) | 4.5 (3.1, 5.8) | 3.7 (3.2, 4.9) | 4.7 (3.7, 6.6) | **0.005**** |
| **PRL (ng/mL)** | 14.8 (12.0, 18.6) | 15.5 (12.4, 18.3) | 13.5 (9.7, 17.4) | 16.2 (11.3, 21.0) | 0.486 |
| **T (ng/mL)** | 0.3 (0.2, 0.4) | 0.2 (0.2, 0.3) | 0.2 (0.1, 0.2) | 0.2 (0.1, 0.3) | 0.126 |
| **Endometrial preparation program** |  |  |  |  | 0.880 |
| Natural cycle | 61.4% (27/44) | 54.4% (31/57) | 56.3% (9/16) | 59.0% (118/200) |  |
| Hormone replacement therapy cycle | 38.6% (17/44) | 45.6% (26/57) | 43.8% (7/16) | 41.0% (82/200) |  |
| **The percentage of RIF/RM/Others** |  |  |  |  | **< 0.001***** |
| RIF | 36.4% (16/44) | 38.6% (22/57) | 25.0% (4/16) | 16.5% (33/200) |  |
| RM | 11.4% (5/44) | 17.5% (10/57) | 37.5% (6/16) | 46.0% (92/200) |  |
| Others | 52.3% (23/44) | 43.9% (25/57) | 37.5% (6/16) | 37.5% (75/200) |  |
| **Duration of post-operative period (years)** | 3.5 (2.0, 5.8) | 3.0 (1.9, 5.0) | 2.0 (1.5, 6.5) | / |  |

Note: BMI: body mass index; E2: estradiol; P: progesterone; FSH: follicle-stimulating hormone; LH: luteinizing hormone; PRL: prolactin; T: testosterone; RIF: repeated implantation failure; RM: recurrent miscarriage; Others: reproductive failure patients who failed to achieve a pregnancy or experienced miscarriage but not meeting the standard of RIF or RM; Mixed endometriosis: endometriotic lesions within both the pelvis and ovary.

Serum hormone levels were all detected on day 3 of the menstrual cycle.

Continuous variables without normal distribution: Kruskal-Wallis test, and shown as median (interquartile range); categorical variables: Chi-square test, shown as percentages of participants.

E2: Ovarian endometriosis versus Control, *P* = 0.003.

LH: Ovarian endometriosis versus Control, *P* = 0.023.

The percentage of RIF: Ovarian endometriosis versus Control, *P* = 0.002; Pelvic endometriosis versus Control, *P* < 0.001.

The percentage of RM: Ovarian endometriosis versus Control, *P* < 0.001; Pelvic endometriosis versus Control, *P* < 0.001.

The percentage of Others: Ovarian endometriosis versus Control, *P* = 0.040.

**：*P* < 0.01; ***: *P* < 0.001.

**Supplementary Table S3** Baseline characteristics in the “Others” population.

|  | **Endometriosis (n = 54)** | **Control (n = 75)** | ***P*-value** |
| --- | --- | --- | --- |
| **Age (years)** | 33.0 (30.8, 36.0) | 34.0 (31.0, 37.0) | 0.330 |
| **BMI (kg/m^2^)** | 21.3 ± 2.7 | 21.8 ± 3.0 | 0.339 |
| **E2 (pg/mL)** | 39.8 (30.8, 53.4) | 28.0 (22.0, 45.6) | **0.006**** |
| **P (ng/mL)** | 0.3 (0.2, 0.4) | 0.4 (0.3, 0.5) | 0.052 |
| **FSH (IU/L)** | 6.8 (5.6, 7.7) | 7.0 (6.1, 8.4) | 0.320 |
| **LH (IU/L)** | 4.1 (2.7, 5.4) | 4.7 (3.6, 6.9) | **0.011*** |
| **PRL (ng/mL)** | 15.2 (12.7, 20.3) | 17.0 (11.3, 21.0) | 0.684 |
| **T (ng/mL)** | 0.3 (0.1, 0.4) | 0.2 (0.1, 0.3) | 0.403 |
| **Endometrial preparation program** |  |  | 0.137 |
| Natural cycle | 48.1% (26/54) | 61.3% (46/75) |  |
| Hormone replacement therapy cycle | 51.9% (28/54) | 38.7% (29/75) |  |
| **Duration of post-operative period (years)** | 3.0 (2.0, 4.5) | / |  |

**Supplementary Table** **S4** Immune responses of the “Others” population.

|  | **Endometriosis (n = 54)** | **Control (n = 75)** | ***P*-value** |
| --- | --- | --- | --- |
| **eCD56^+^ (%)** | 10.7 (7.2, 16.5) | 12.2 (8.7, 20.6) | 0.104 |
| **eCD68^+^ (%)** | 2.0 (1.4, 2.9) | 2.2 (1.6, 2.8) | 0.551 |
| **eCD1a^+^ (%)** | 0.06 (0.03, 0.10) | 0.06 (0.03, 0.12) | 0.599 |
| **eCD83^+^ (%)** | 1.6 (1.0, 2.4) | 1.8 (1.2, 2.9) | 0.317 |
| **eCD57^+^ (%)** | 0.4 (0.2, 0.5) | 0.3 (0.2, 0.6) | 0.753 |
| **eCD8^+^ (%)** | 3.0 (2.0, 3.9) | 3.1 (2.0, 4.4) | 0.532 |
| **eFoxP3^+^ (%)** | 0.08 (0.05, 0.11) | 0.10 (0.06, 0.16) | 0.069 |
| **pCD3^+^ (%)** | 70.5 ± 6.8 | 67.7 ± 8.8 | 0.067 |
| **pCD3^+^CD8^+^ (%)** | 26.7 (23.5, 31.7) | 25.3 (22.5, 28.1) | 0.055 |
| **pCD3^+^CD4^+^ (%)** | 36.2 ± 5.8 | 35.4 ± 7.0 | 0.518 |
| **pCD56^+^CD16^+^ (%)** | 15.2 ± 6.0 | 17.6 ± 8.4 | 0.066 |
| **pCD19^+^ (%)** | 12.0 (9.9, 15.8) | 12.5 (10.5, 15.2) | 0.705 |
| **Cytotoxicity at ET25 (%)** | 19.9 (15.8, 29.4) | 35.6 (23.9, 43.6) | **< 0.001***** |
| **Cytotoxicity at ET50 (%)** | 32.6 (21.5, 39.6) | 48.3 (33.7, 53.9) | **< 0.001***** |

**Supplementary Table S5** Baseline characteristics in the RIF population.

|  | **Endometriosis (n = 42)** | **Control (n = 33)** | ***P*-value** |
| --- | --- | --- | --- |
| **Age (years)** | 34.9 ± 4.1 | 35.9 ± 5.2 | 0.368 |
| **BMI (kg/m^2^)** | 20.3 ± 2.0 | 21.5 ± 3.0 | **0.033*** |
| **E2 (pg/mL)** | 39.6 ± 15.4 | 43.6 ± 20.1 | 0.348 |
| **P (ng/mL)** | 0.4 (0.2, 0.6) | 0.4 (0.2, 0.6) | 0.803 |
| **FSH (IU/L)** | 7.4 (6.0, 9.3) | 7.3 (6.0, 8.6) | 0.610 |
| **LH (IU/L)** | 4.2 (3.0, 5.3) | 4.7 (3.7, 6.5) | **0.040*** |
| **PRL (ng/mL)** | 15.6 ± 5.0 | 16.2 ± 4.4 | 0.587 |
| **T (ng/mL)** | 0.2 (0.2, 0.3) | 0.3 (0.1, 0.4) | 0.538 |
| **Endometrial preparation program** |  |  | 0.482 |
| Natural cycle | 61.9% (26/42) | 69.7% (23/33) |  |
| Hormone replacement therapy cycle | 38.1% (16/42) | 30.3% (10/33) |  |
| **Duration of post-operative period (years)** | 4.0 ± 2.7 | / |  |

**Supplementary Table S6** Immune responses of the RIF population.

|  | **Endometriosis (n = 42)** | **Control (n = 33)** | ***P*-value** |
| --- | --- | --- | --- |
| **eCD56^+^ (%)** | 14.0 (8.8, 21.2) | 13.1 (9.8, 20.3) | 0.991 |
| **eCD68^+^ (%)** | 2.5 ± 1.1 | 2.3 ± 1.3 | 0.498 |
| **eCD1a^+^ (%)** | 0.06 (0.04, 0.10) | 0.06 (0.03, 0.12) | 0.543 |
| **eCD83^+^ (%)** | 1.9 (1.3, 2.4) | 1.9 (1.3, 2.3) | 0.818 |
| **eCD57^+^ (%)** | 0.4 (0.2, 0.5) | 0.4 (0.2, 0.5) | 0.974 |
| **eCD8^+^ (%)** | 2.9 (2.3, 3.9) | 2.8 (2.0, 4.1) | 0.839 |
| **eFoxP3^+^ (%)** | 0.11 (0.07, 0.15) | 0.08 (0.06, 0.12) | 0.055 |
| **pCD3^+^ (%)** | 68.0 ± 6.7 | 68.2 ± 7.2 | 0.882 |
| **pCD3^+^CD8^+^ (%)** | 25.2 ± 5.9 | 26.0 ± 7.6 | 0.630 |
| **pCD3^+^CD4^+^ (%)** | 37.7 ± 6.4 | 35.7 ± 6.7 | 0.201 |
| **pCD56^+^CD16^+^ (%)** | 17.6 ± 7.3 | 17.0 ± 7.7 | 0.730 |
| **pCD19^+^ (%)** | 13.2 ± 4.0 | 13.6 ± 3.4 | 0.627 |
| **Cytotoxicity at ET25 (%)** | 29.0 ± 14.4 | 32.4 ± 15.5 | 0.332 |
| **Cytotoxicity at ET50 (%)** | 39.1 ± 15.2 | 42.1 ± 16.1 | 0.407 |

**Supplementary Table S7** Baseline characteristics in the RM population.

|  | **Endometriosis (n = 21)** | **Control (n = 92)** | ***P*-value** |
| --- | --- | --- | --- |
| **Age (years)** | 35.7 ± 4.1 | 33.9 ± 5.0 | 0.119 |
| **BMI (kg/m^2^)** | 21.0 ± 2.2 | 21.7 ± 2.8 | 0.302 |
| **E2 (pg/mL)** | 36.8 (27.2, 52.2) | 31.4 (25.0, 43.7) | 0.122 |
| **P (ng/mL)** | 0.3 (0.2, 0.6) | 0.4 (0.3, 0.6) | 0.306 |
| **FSH (IU/L)** | 7.4 (6.0, 9.0) | 6.7 (5.5, 8.2) | 0.132 |
| **LH (IU/L)** | 4.6 (3.7, 5.9) | 4.7 (3.7, 6.4) | 0.726 |
| **PRL (ng/mL)** | 13.2 (10.9, 17.2) | 15.0 (11.1, 21.7) | 0.349 |
| **T (ng/mL)** | 0.2 (0.2, 0.3) | 0.2 (0.2, 0.4) | 0.973 |
| **Endometrial preparation program** |  |  | 0.153 |
| Natural cycle | 71.4% (15/21) | 54.3% (50/92) |  |
| Hormone replacement therapy cycle | 28.6% (6/21) | 45.7% (42/92) |  |
| **Duration of post-operative period (years)** | 4.6 ± 2.9 | / |  |

**Supplementary Table S8** Immune responses of the RM population.

|  | **Endometriosis (n = 21)** | **Control (n = 92)** | ***P*-value** |
| --- | --- | --- | --- |
| **eCD56^+^ (%)** | 13.8 (7.6, 20.1) | 11.9 (8.1, 16.8) | 0.595 |
| **eCD68^+^ (%)** | 2.4 ± 0.8 | 2.1 ± 0.9 | 0.133 |
| **eCD1a^+^ (%)** | 0.07 (0.03, 0.10) | 0.05 (0.03, 0.09) | 0.150 |
| **eCD83^+^ (%)** | 1.9 (1.4, 2.4) | 1.6 (1.1, 2.3) | 0.271 |
| **eCD57^+^ (%)** | 0.4 (0.3, 0.6) | 0.4 (0.2, 0.5) | 0.400 |
| **eCD8^+^ (%)** | 3.0 ± 1.5 | 3.2 ± 1.7 | 0.606 |
| **eFoxP3^+^ (%)** | 0.13 (0.08, 0.17) | 0.10 (0.06, 0.13) | **0.021*** |
| **pCD3^+^ (%)** | 70.0 ± 7.1 | 69.1 ± 8.1 | 0.676 |
| **pCD3^+^CD8^+^ (%)** | 25.5 ± 5.7 | 27.4 ± 6.8 | 0.224 |
| **pCD3^+^CD4^+^ (%)** | 38.0 ± 7.6 | 36.1 ± 6.6 | 0.248 |
| **pCD56^+^CD16^+^ (%)** | 15.9 (13.2, 20.7) | 16.7 (11.9, 20.2) | 0.788 |
| **pCD19^+^ (%)** | 12.0 ± 3.1 | 12.7 ± 3.9 | 0.438 |
| **Cytotoxicity at ET25 (%)** | 28.8 ± 17.6 | 32.5 ± 13.8 | 0.294 |
| **Cytotoxicity at ET50 (%)** | 37.4 (20.7, 54.9) | 44.5 (33.2, 51.8) | 0.136 |

Note: e: endometrial cell; p: peripheral blood mononuclear cell.

Cytotoxicity: cytotoxicity of peripheral NK cells, shown as the ratios of target cell lysis. ET: the ratios between effectors and target cells.

Continuous variables with normal distribution: t-test, and shown as mean ± standard deviation; Continuous variables without normal distribution: Mann–Whitney U test, and shown as median (interquartile range).

*: *P* < 0.05; **: *P* < 0.01; ***：*P* < 0.001.

**Supplementary Table S9** Correlation analysis.

|  |  | **The whole study population** | | | | | | **Endometriosis group** | | | | | | **Control group** | | | | | |
| --- | --- | --- | --- | --- | --- | --- | --- | --- | --- | --- | --- | --- | --- | --- | --- | --- | --- | --- | --- |
|  |  | **E2** | **LH** | **BMI** | **eCD68** | **ET25** | **ET50** | **E2** | **LH** | **BMI** | **eCD68** | **ET25** | **ET50** | **E2** | **LH** | **BMI** | **eCD68** | **ET25** | **ET50** |
| **E2** | Pearson correlation | 1 | 0.020 | -0.107 | **.150^**^** | -0.048 | -0.047 | 1 | -0.065 | 0.078 | **.251^**^** | -0.065 | -0.053 | 1 | 0.089 | -.169^*^ | **0.084** | -0.019 | -0.021 |
|  | Sig.（two-tail） |  | 0.722 | 0.060 | **0.008** | 0.408 | 0.416 |  | 0.505 | 0.419 | **0.009** | 0.506 | 0.586 |  | 0.212 | 0.017 | **0.238** | 0.794 | 0.768 |
| **LH** | Pearson correlation | 0.020 | 1 | -.118^*^ | -0.062 | -0.047 | -0.063 | -0.065 | 1 | -0.065 | -0.092 | 0.016 | 0.021 | 0.089 | 1 | -.180^*^ | -0.027 | -0.131 | -.162^*^ |
|  | Sig.（two-tail） | 0.722 |  | 0.039 | 0.280 | 0.412 | 0.270 | 0.505 |  | 0.501 | 0.340 | 0.871 | 0.833 | 0.212 |  | 0.011 | 0.703 | 0.064 | 0.022 |
| **BMI** | Pearson correlation | -0.107 | -.118^*^ | 1 | -0.047 | 0.009 | 0.031 | 0.078 | -0.065 | 1 | -0.025 | -0.017 | 0.000 | -.169^*^ | -.180^*^ | 1 | -0.040 | -0.027 | -0.005 |
|  | Sig.（two-tail） | 0.060 | 0.039 |  | 0.409 | 0.868 | 0.591 | 0.419 | 0.501 |  | 0.793 | 0.863 | 0.999 | 0.017 | 0.011 |  | 0.570 | 0.701 | 0.947 |
| **eCD68** | Pearson correlation | .150^**^ | -0.062 | -0.047 | 1 | -0.038 | -0.063 | .251^**^ | -0.092 | -0.025 | 1 | -0.064 | -0.055 | 0.084 | -0.027 | -0.040 | 1 | -0.001 | -0.045 |
|  | Sig.（two-tail） | 0.008 | 0.280 | 0.409 |  | 0.507 | 0.267 | 0.009 | 0.340 | 0.793 |  | 0.504 | 0.560 | 0.238 | 0.703 | 0.570 |  | 0.991 | 0.526 |
| **ET25** | Pearson correlation | -0.048 | -0.047 | 0.009 | -0.038 | 1 | .950^**^ | -0.065 | 0.016 | -0.017 | -0.064 | 1 | .955^**^ | -0.019 | -0.131 | -0.027 | -0.001 | 1 | .943^**^ |
|  | Sig.（two-tail） | 0.408 | 0.412 | 0.868 | 0.507 |  | 0.000 | 0.506 | 0.871 | 0.863 | 0.504 |  | 0.000 | 0.794 | 0.064 | 0.701 | 0.991 |  | 0.000 |
| **ET50** | Pearson correlation | -0.047 | -0.063 | 0.031 | -0.063 | .950^**^ | 1 | -0.053 | 0.021 | 0.000 | -0.055 | .955^**^ | 1 | -0.021 | -.162^*^ | -0.005 | -0.045 | .943^**^ | 1 |
|  | Sig.（two-tail） | 0.416 | 0.270 | 0.591 | 0.267 | 0.000 |  | 0.586 | 0.833 | 0.999 | 0.560 | 0.000 |  | 0.768 | 0.022 | 0.947 | 0.526 | 0.000 |  |

Note: E2: baseline estradiol; LH: baseline luteinizing hormone; BMI: body mass index; e: endometrial cell; ET: cytotoxicity of peripheral NK cells, shown as the ratios between effectors and target cells; *: *P* < 0.05; **: *P* < 0.001.

# Supplementary Figures


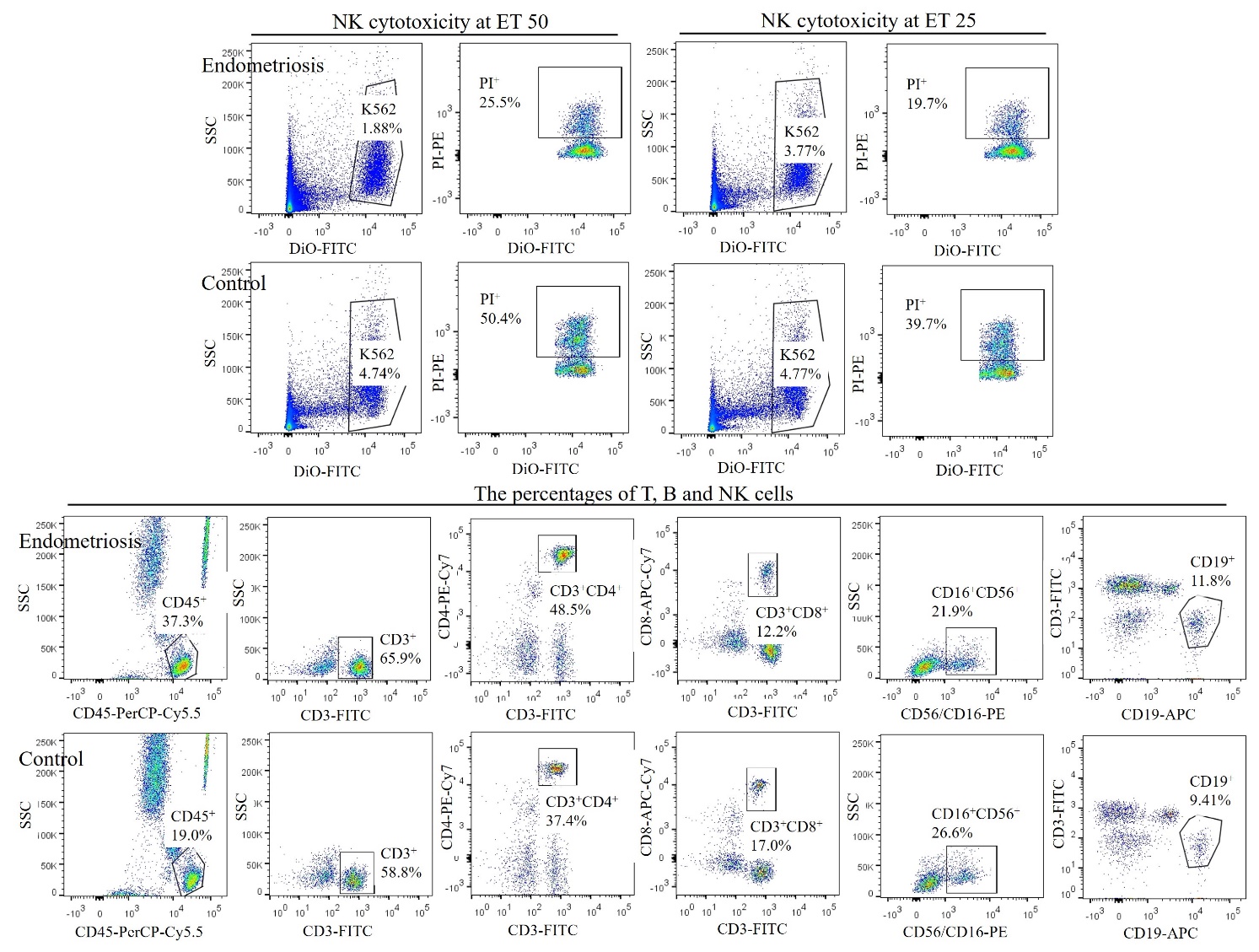


**Supplementary Figure S1.** Representative images of peripheral NK cytotoxicity and lymphocyte subsets in the study population.


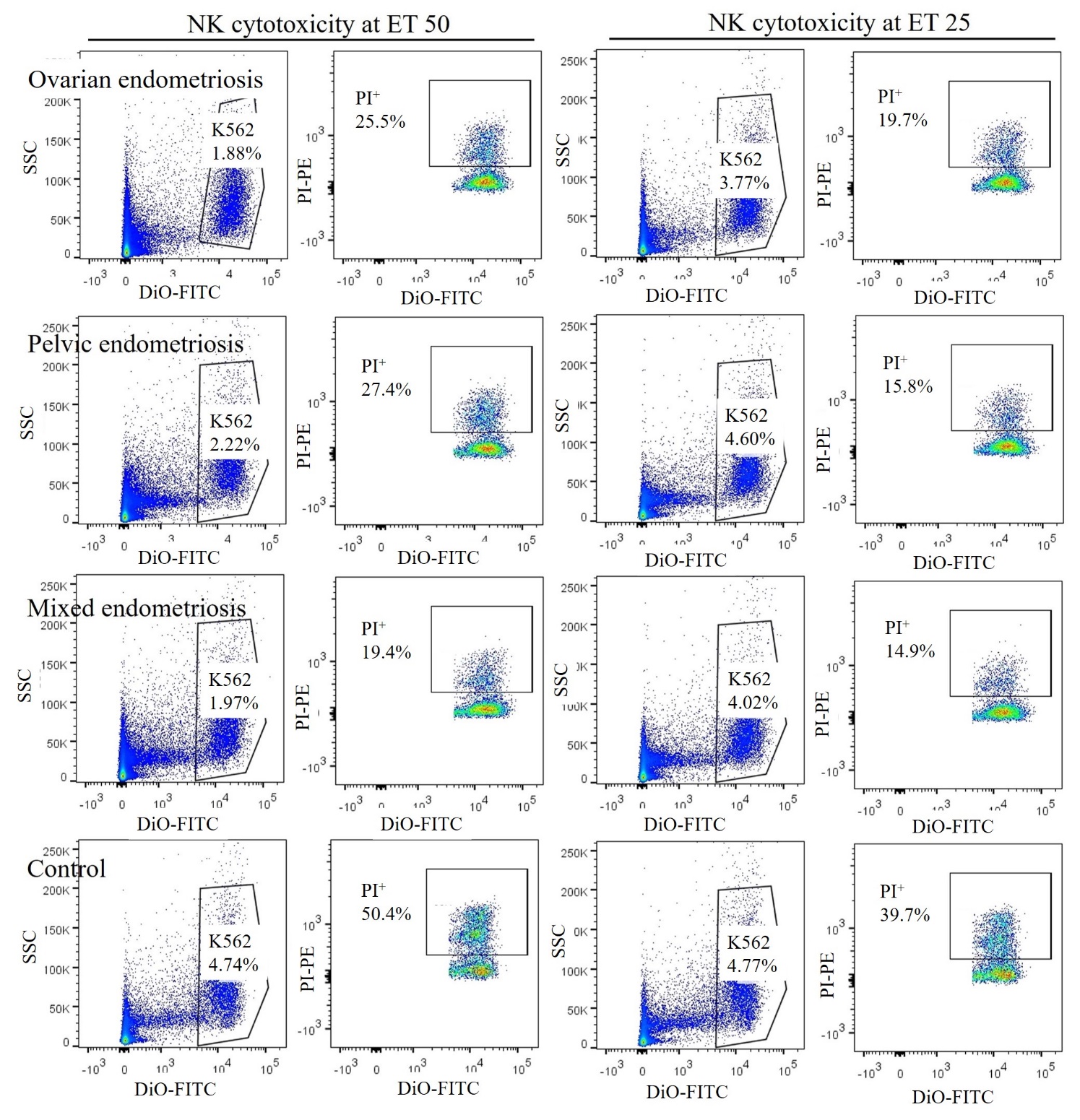


**Supplementary Figure S2.** Representative images of peripheral NK cytotoxicity in the subgroups.


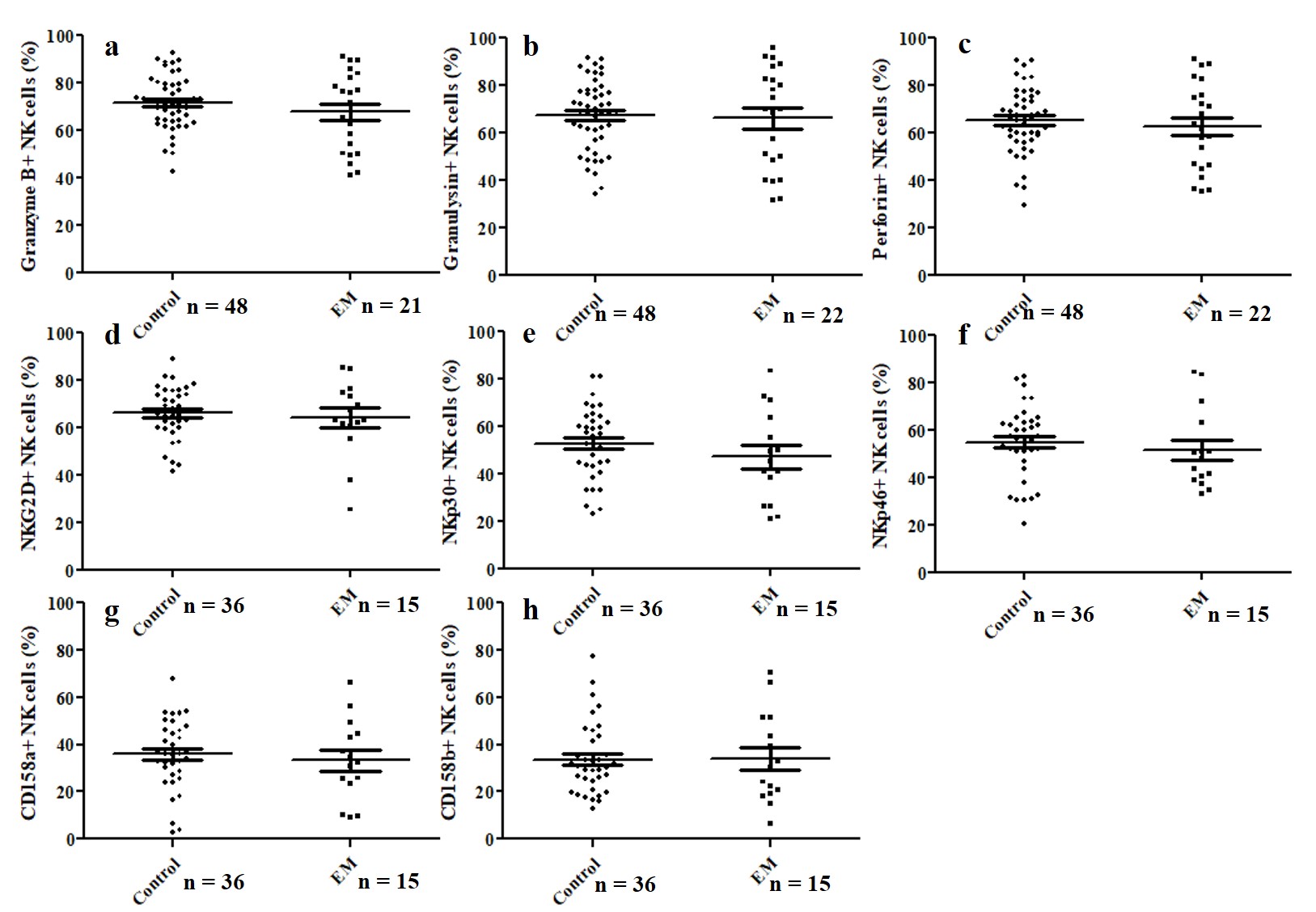


**Supplementary Figure S3.** Expression of intracellular immune mediators of peripheral NK cells and expression of NK cell surface receptors of part of the study population. Comparison of the expression of granzyme B (a), granulysin (b), perforin (c), NKG2D (d), NKp30 (e), NKp46 (f), CD158a (g), and CD158b (h) of peripheral NK cells between non-endometriosis and endometriosis groups.


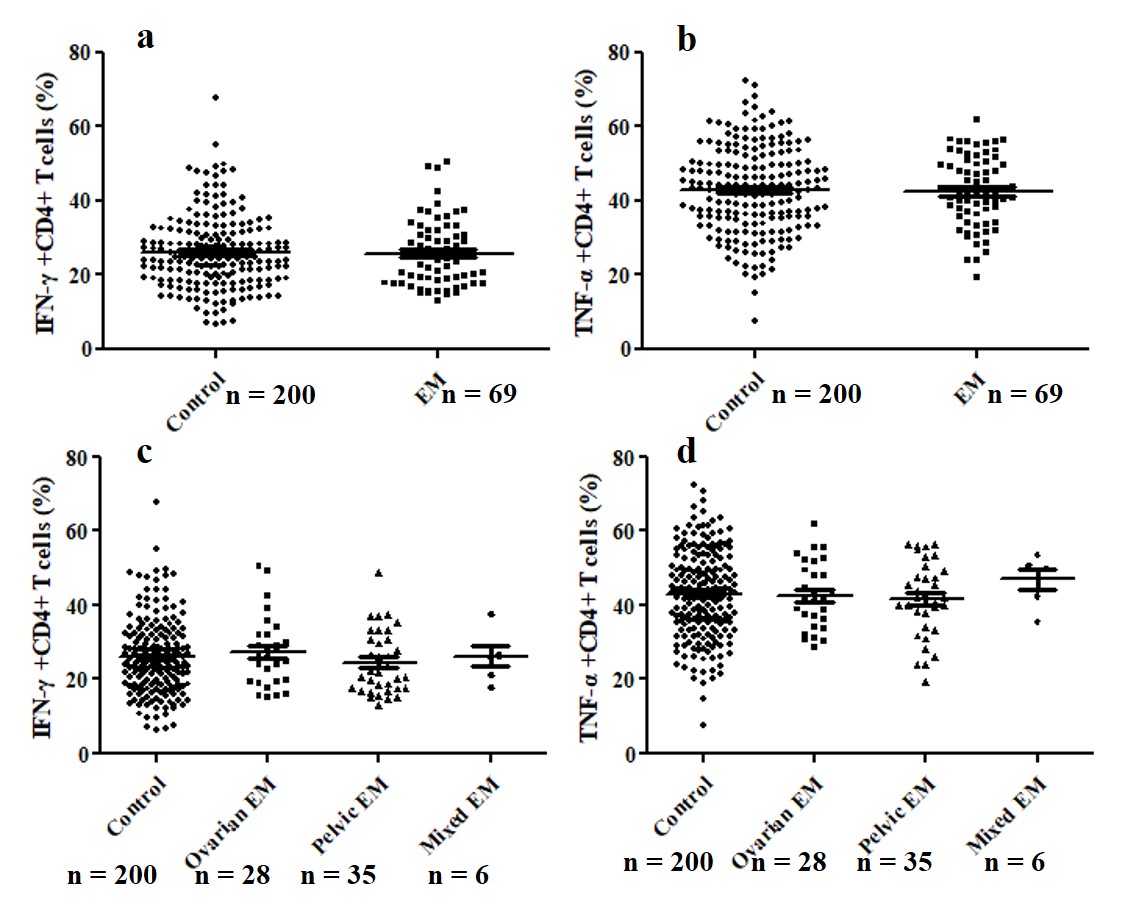


**Supplementary Figure S4** Production of IFN-γ and TNF-α by peripheral CD4^+^ T cells in part of the study population. Expressions of IFN-γ and TNF-α were similar between patients with or without endometriosis (a, b) and similar among the subgroups (c, d).


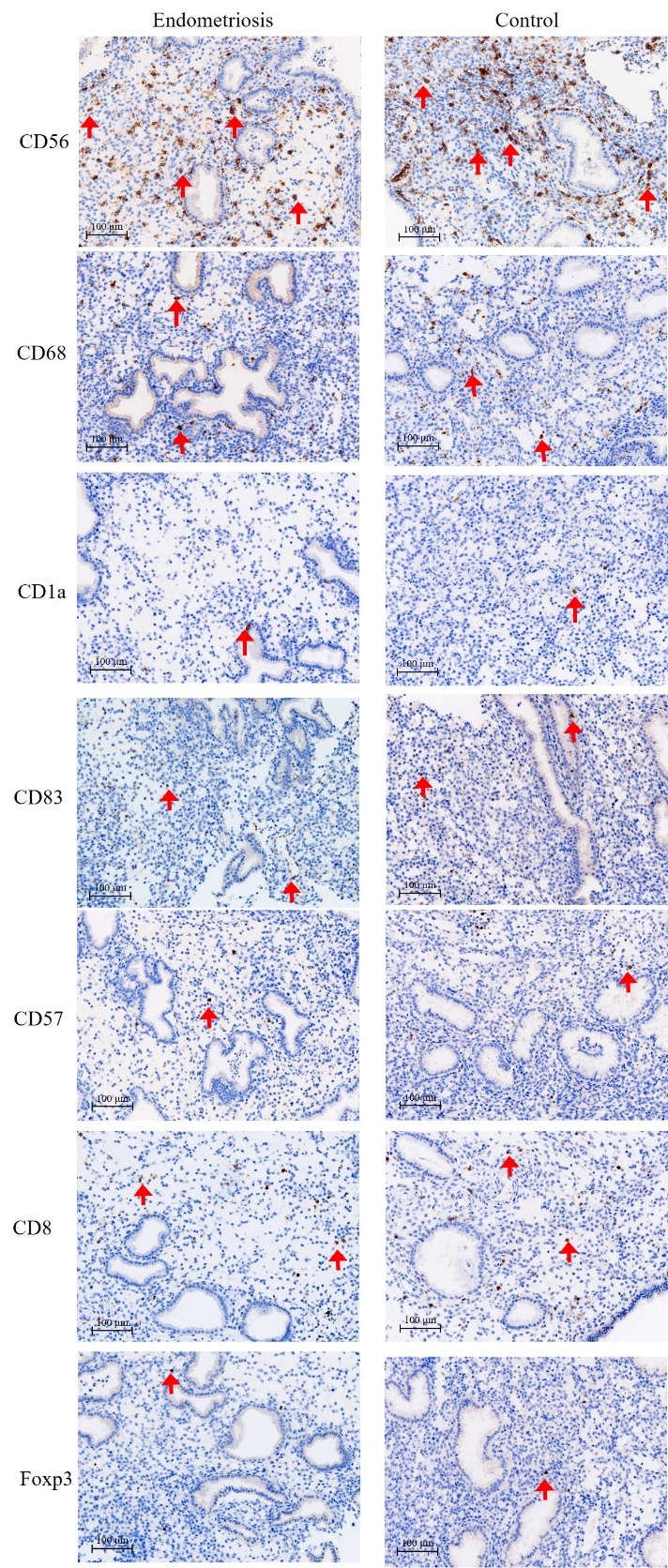


**Supplementary Figure S5.** Representative images of uterine immune cells in the study population. Classical endometriotic immune cells are marked with red arrows. Magnification: 200 X.


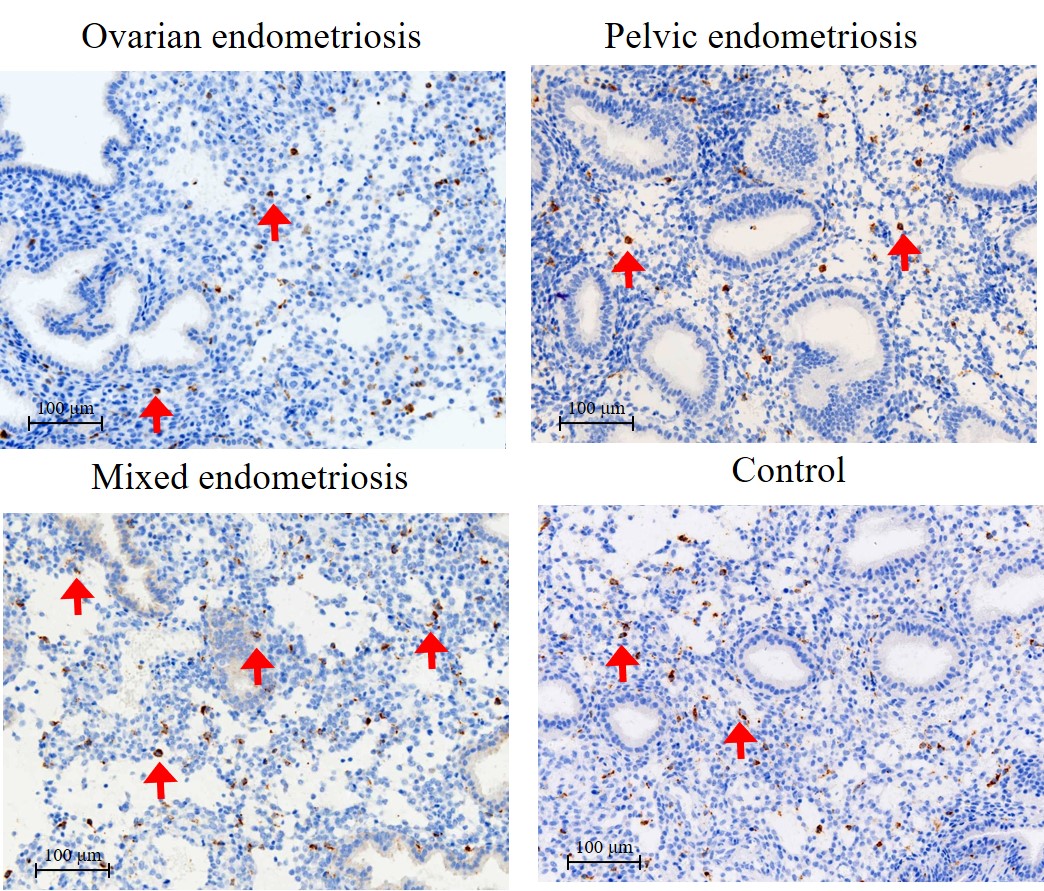


**Supplementary Figure S6.** Representative images of uterine CD68^+^ macrophages in the subgroups. Classical CD68^+^ macrophages are marked with red arrows. Magnification: 200 X.


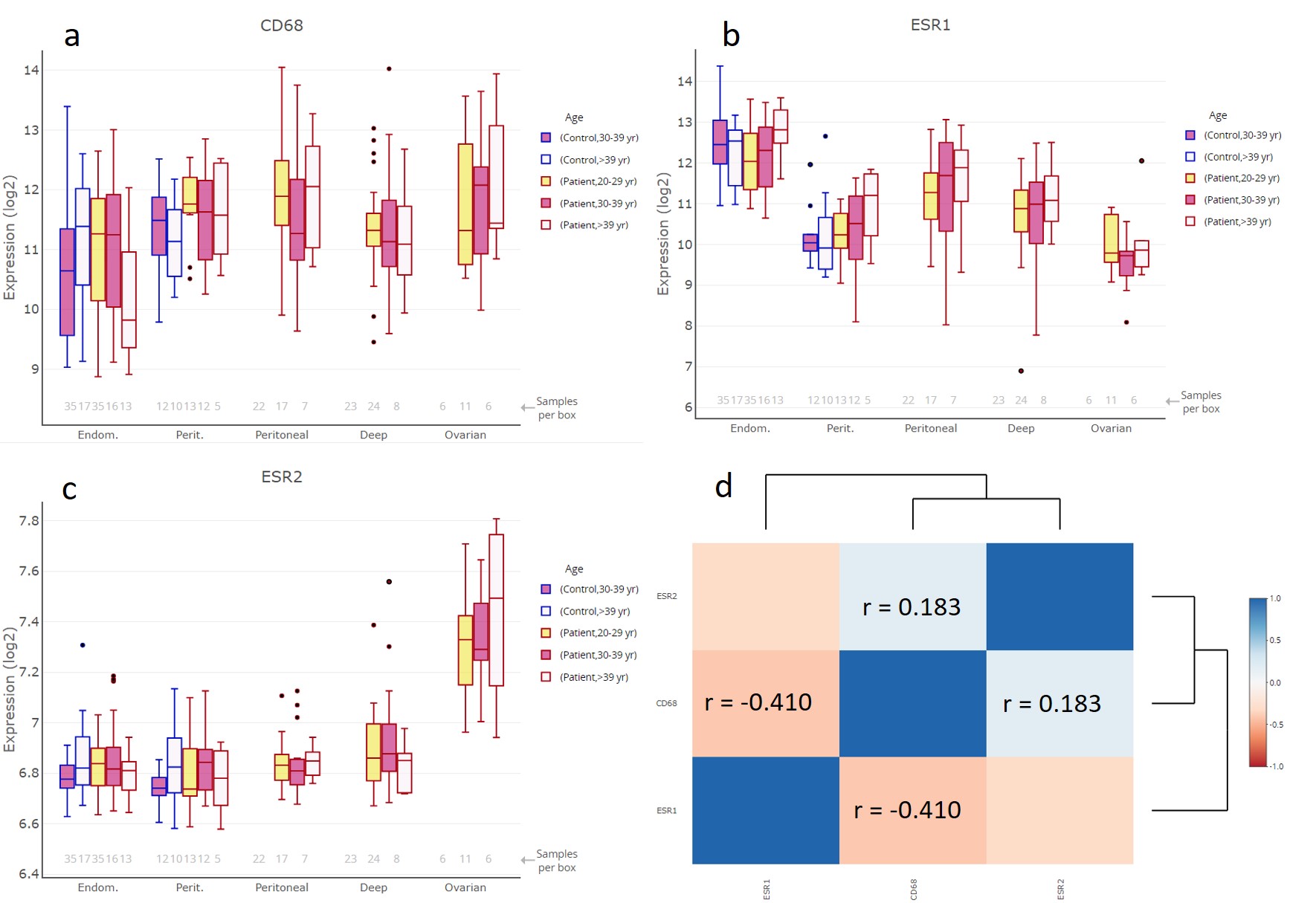


**Supplementary Figure S7.** mRNA expression of ESR1, ESR2, and CD68 in the endometrium and peritoneum of the control group, and in the eutopic and ectopic endometria and peritoneum of patients with endometriosis in EndometDB, as well as their correlation. (a) mRNA expression of CD68; (b) mRNA expression of ESR1; (c) mRNA expression of ESR2; (d) correlation between ESR1, ESR2, and CD68 in the study population.
